# Investigating young adults’ mental health and early working life trajectories from a life course perspective: the role of transitions

**DOI:** 10.1136/jech-2019-213245

**Published:** 2019-11-06

**Authors:** Ute Bültmann, Iris Arends, Karin Veldman, Christopher B. McLeod, Sander K.R. van Zon, Benjamin C. Amick III

**Affiliations:** 1 Department of Health Sciences, Community and Occupational Medicine, University Medical Center Groningen, University of Groningen, Groningen, The Netherlands; 2 School of Population and Public Health, The University of British Columbia, Vancouver, British Columbia, Canada; 3 Institute for Work & Health, Toronto, Ontario, Canada; 4 Department of Health Policy and Management, Robert Stempel College of Public Health and Social Work, Florida International University, Miami, Florida, USA

**Keywords:** mental health, life course epidemiology, lifecourse/childhood circumstances, occupational health

## Abstract

**Background:**

Many young adults leave the labour market because of mental health problems or never really enter it, through early moves onto disability benefits. Across many countries of the Organisation for Economic Co-operation and Development, between 30% and 50% of all new disability benefit claims are due to mental health problems; among young adults this moves up to 50%–80%.

**Outline:**

We propose a research agenda focused on transitions in building young adults’ mental health and early working life trajectories, considering varying views for subgroups of a society. First, we briefly review five transition characteristics, then we elaborate a research agenda with specific research questions.

**Research agenda:**

Our research agenda focuses on transitions as processes, in time and place and as sensitive periods, when examining young adults’ mental health and early working life trajectories from a life course perspective. As more and more childhood and adolescent cohorts mature and facilitate research on later life labour market, work and health outcomes, transition research can help guide policy and practice interventions.

**Future cross-disciplinary research:**

In view of the many challenges young adults face when entering the changing world of work and labour markets, future research on transitions in young adults related to their mental health and early working life trajectories will provide ample opportunities for collaborative cross-disciplinary research and stimulate debate on this important challenge.

## Research agenda

Young adults, as they enter early working life, experience major life transitions that are embedded in changing social contexts. These transitions—completing school, leaving home, entering the workforce, getting married and having children^1^—are affected by prior mental health and in turn affect future mental health. Evidence is beginning to show the negative mental health consequences of the evolving nature of work.^2 3^

Many young adults leave the labour market because of mental health problems or never really enter it, through early moves onto disability benefits.^4^ Across many countries of the Organisation for Economic Co-operation and Development, between 30% and 50% of all new disability benefit claims are due to mental health problems; among young adults this moves up to 50%–80%.^4–6^ However, research on mental health and work remains fragmented, focusing on specific exposures at specific time points and/or occupational groups. Incorporating a life course perspective is necessary for understanding the contribution of prework mental health experiences on working life opportunities and later life mental health problems.

In a 2015 editorial, Viner and colleagues^7^ advanced a life course epidemiology research agenda that recognised the significance of adolescence as both a sensitive or critical exposure period for later life health and disease. Surprisingly, the authors do not mention ‘work’ in their note on future life course epidemiology research. We consider it important to link mental health in adolescence not only to later life health and disease^7^ but also to later life education and work outcomes.^8–10^

In an earlier paper, an integrated life course perspective for building working life trajectories was described.^11^ While recognising the importance of transitions,^11^ we consider it relevant to more deeply elaborate on transitions as processes in time and place, as sensitive or critical periods, and as contributing to the accumulation of risk/benefit, to help better identify policy and practice interventions in early working life. Transitions are core to life course research and not only stem from sociology and psychology (eg, refs 12 13) but also from demography, biology, economic and political sciences (eg, refs 14 15). For the integration of a life course perspective in the intersecting research field of ‘work and mental health among young adults’, a transdisciplinary approach is needed that draws on occupational and youth/community mental health, epidemiological, sociological, psychological and health economics research, and that further brings in fields where economic, family and social welfare policy are important action levers.

We propose a detailed research agenda focused on transitions in building young adults’ mental health and early working life trajectories, considering varying views for societal subgroups defined by gender, social class, ethnicity, culture or by demographic cohort.^16^ First, we briefly review five transition characteristics, then we elaborate a research agenda with specific research questions.

1. Transitions should be considered as a process not a change in state.
2. Transitions are defined by time and place.
3. Transitions may be sensitive or critical periods.
4. Transitions contribute to the accumulation of (health) risk and benefits.
5. Transitions are core to building trajectories.

### Transition as a process not a change in state

Defining a transition as a change from one state to another disregards the dynamics of transitions.^17 18^ Often the boundaries of a transition are not known. Consider the school to work transition where a first job is often seen as the transition end point, while it may be the starting point of the transition process. A first job is frequently unstable and temporary, and it often takes some time to find a job that offers stability and career prospects. Treating a transition as a change in one single status will offer very limited and possibly inaccurate information for policy and practice (eg, focusing on helping young adults into any kind of first job). Moving research forward calls for new definitions of transitions, stepping away from single status change to transition periods where multiple states can occur, reoccur and co-occur within different contexts.^17^

### Transitions are defined by time and place

Transitions as a process are time-dependent and place-dependent. Age, period and cohort effects are important time-variant elements to consider when investigating transitions. For example, tight labour market conditions at graduation age affect the labour market entrance of young adults and have persistent negative effects on earnings, employment and disability pension utilisation.^19^ Similarly, place, for example, the regional or national structure of labour markets or the type of social welfare system, can shape the transition process. Earlier research has shown how welfare regimes shape transitions of youth to work^20 21^ or the employment behaviour of women.^22^ For example, regimes with more family-friendly policies (public childcare, parental leave arrangements) support women’s employment behaviour.^22^ However, the timing of mental health in individual life courses and the role of mental health management in welfare regimes have not been investigated when looking at young adults’ transition processes into work.

### Transitions may be sensitive (or critical) periods

Transitions may be sensitive periods where exposures are exacerbated or ameliorated. A person experiencing depressive symptoms during the transition period into the labour market may experience more or less detrimental work and mental health consequences across the life course than a person experiencing depressive symptoms later in life.^23 24^ Because sensitive periods represent periods in which policies and practices may be most effective, the identification of sensitive periods is a key research priority. Dunn and colleagues^25^ applied life course models, such as sensitive periods, to explain the relation between childhood adversity and psychopathology symptoms.^25^ Emphasising that sensitive periods for mental health problems during childhood and adolescence may affect labour market participation of young adults guides our research towards transitions as sensitive periods.

### Transitions may contribute to the accumulation of (health) risk and benefits

Understanding the dynamics of transitions requires a clearer specification of accumulating (health) risks and benefits during transitions. To date, many young adults are in unstable, informal employment, or unable to get jobs.^26^ When young adults transition into poor working conditions and are exposed for some time, (new) risks may emerge that accumulate or cluster with adverse consequences for work and mental health in later life. In many countries, school to work transitions are now slower with limited job opportunities.^27^ Yet little is known about the possible mental health benefits of transitioning into new labour markets and the accumulation of risk from different working and non-working states.

### Transitions are core to building trajectories

Transitions allow investigating important change processes embedded in larger contexts that, together, form life course trajectories.^28 29^ The little research available on working life trajectories uses the sequencing of changes in labour market outcomes/states, for example, from employment to unemployment,^30–32^ and links the trajectories to mental health outcomes. However, studying only changes in labour market states overlooks how transition processes are shaped by contextual influences. In the example of the school to work transition, the process towards stable employment will be different in a tight labour market or with the economic security of a good-earning partner. Such contextual influences are inherent to the concept of transitions; they shape working life trajectories and affect health outcomes. Research is needed examining transitions and their embeddedness in the larger social, political and economic contexts to adequately understand the relations with mental health outcomes (eg, refs 33 34).

## Proposed research agenda

To guide future research on transitions in building young adults’ mental health and early working life trajectories, we propose a series of research questions:

1. Does the timing of the school to work transition influence mental health and early working life trajectories of young adults?
2. What are the characteristics of the school to work transition as a dynamic process?
3. What is the influence of mental health in childhood and adolescence on school to work transitions of young adults and how does it differ across regions or countries?
4. How do major life transitions, that is, leaving home, completing school, entering the workforce, getting married and having children, shape the early working life trajectories of young adults?
5. Which biological, psychological and/or social exposures can be identified that exacerbate or ameliorate transitions into or within the labour market?
6. What is the interplay between major life transitions regarding transition timing and duration?
7. How do early work life transitions affect later work life transitions and later life mental health?

The implementation of the above research agenda cannot be achieved by designing a single definitive study, but rather requires building a series of coherent findings from multiple studies that allow synthesising evidence to identify key leverage points for policy and practice. The emerging availability of large multiyear cohorts in multiple jurisdictions provides fertile opportunity for many to investigate the research questions, advance the research field and support evidence-based policy and practice changes. For example, longitudinal qualitative, comparative analysis may help to decompose contextual effects on transition processes. Other methods for life course research include sequence analysis, latent growth trajectories and Markov chain models. Although useful to examine transitions as change of state, the methods may be less appropriate to examine transition processes. Life course methods, focusing on timing, duration and accumulation, may have to be combined and accompanied with longitudinal, qualitative methods to fully capture the dynamic transition processes in building working life trajectories.

We expect answering some questions will lead to new research questions, especially as our understanding of transitions in building young adults’ mental health and early working life trajectories advances. Finally, by focusing the research agenda on the transition into work and the labour market, that is, on a cohort of young workers, we hope to advance our thinking on the working life course without the noise of selection bias introduced when investigating cohorts built in random samples of the population at risk.
